# Supplementary figures and images for: Selective advantages favour high genomic AT-contents in intracellular elements
Source: PLoS Genet. 2019 Apr 29;15(4):e1007778. doi: 10.1371/journal.pgen.1007778 (PMC6519830; doi:10.1371/journal.pgen.1007778)

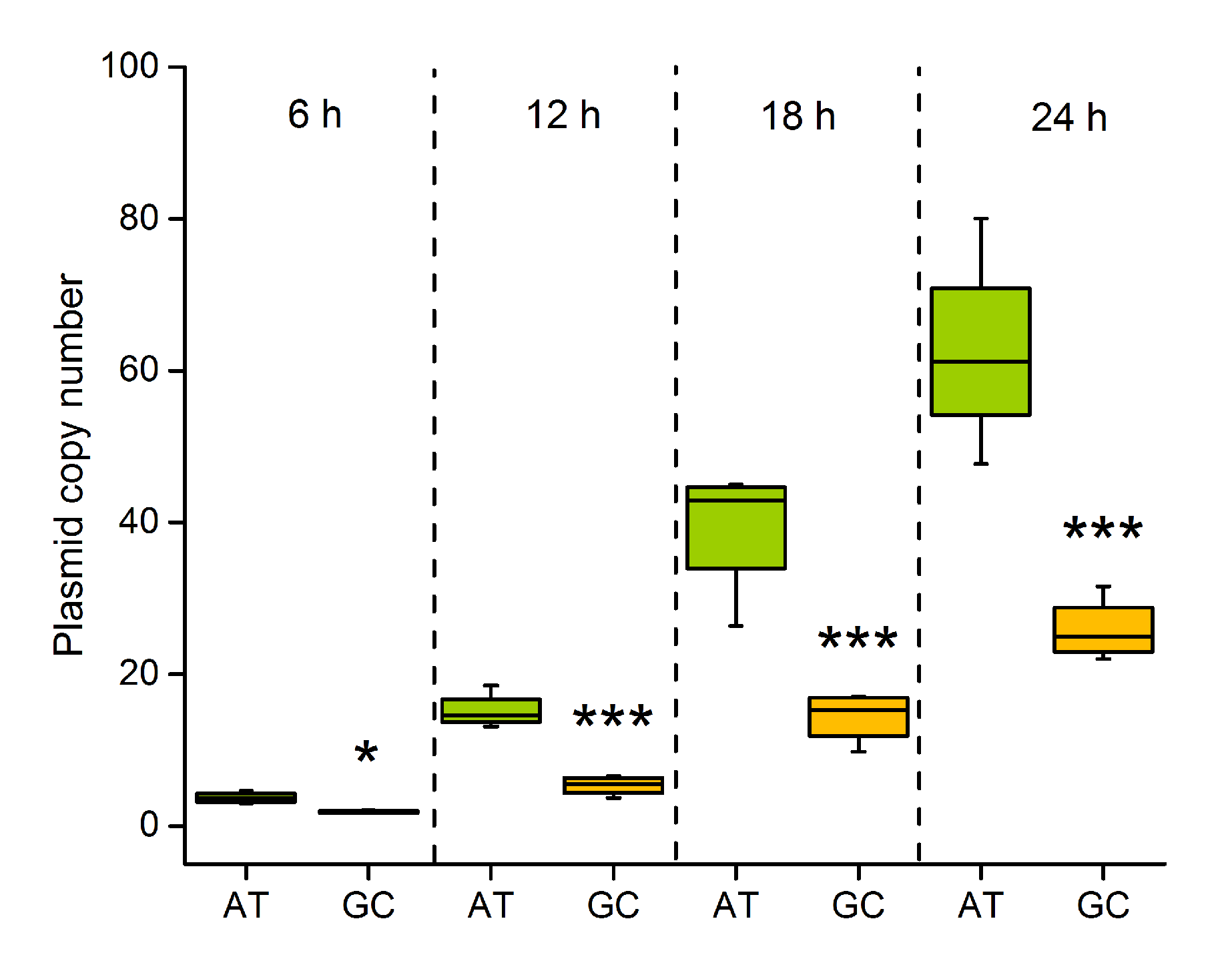

Supplement: S1 Fig — Plasmid copy number per bacterial chromosome equivalent was assessed by quantitative real-time PCR of E. coli cultures at four time points during a 24 h growth period. Shown are copy numbers of pJet plasmids harbouring four randomly chosen AT-rich or GC-rich inserts, respectively. The first two sampling points (i.e. 6 h, 12 h) correspond to the beginning and mid-exponential growth phase, the third sampling point (i.e. 18 h) to the end of exponential growth phase, and the fourth sampling point (i.e. 24 h) to the beginning of stationary growth. Asterisks indicate significant differences between E. coli cells containing AT-rich and GC-rich plasmids within the same time point. Independent-samples t-test: *** P < 0.001, * P < 0.05, n = 4. (TIF) [file pgen.1007778.s001.tif]

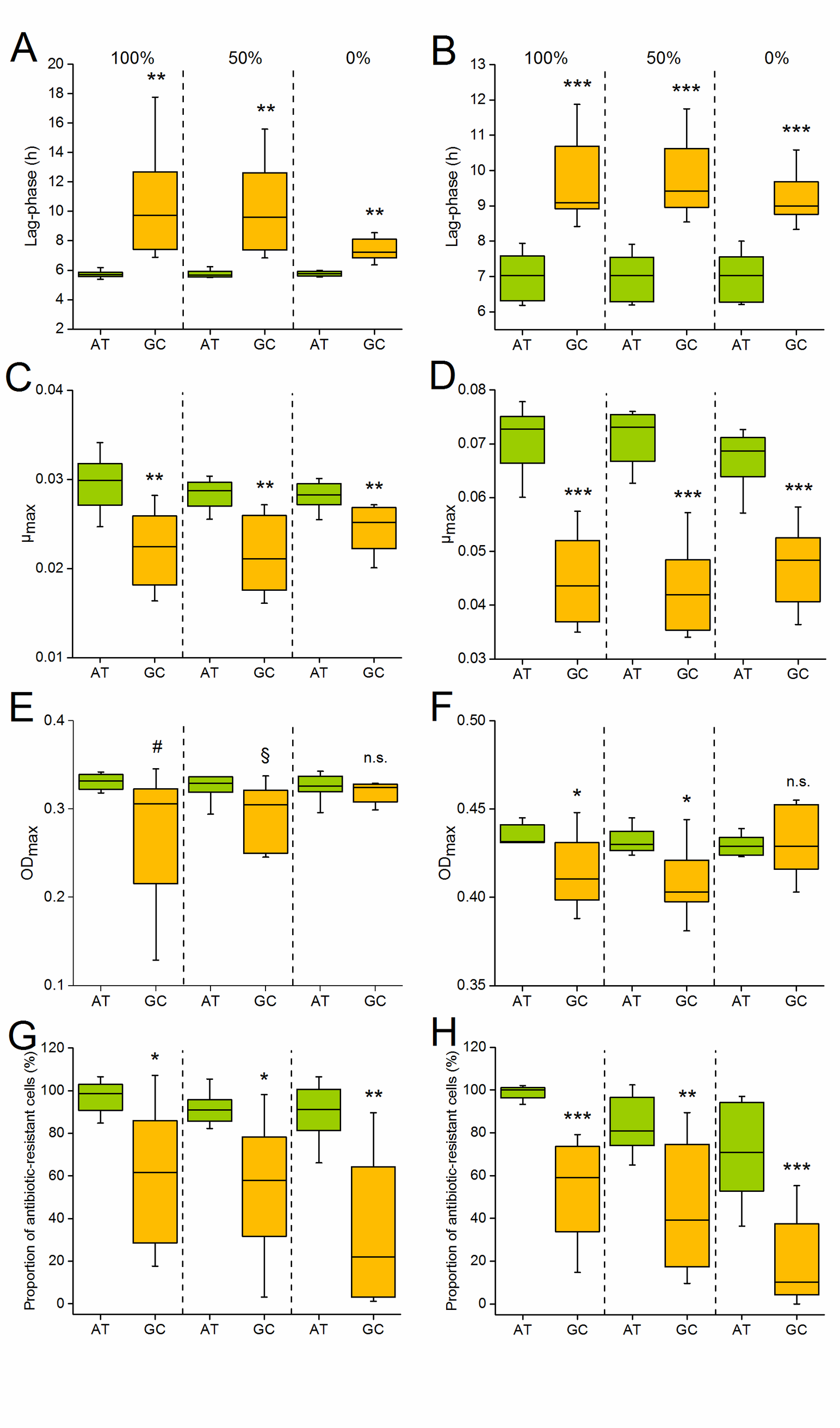

Supplement: S2 Fig — Growth experiments of E. coli harbouring AT-rich (green) or GC-rich (orange) pJet (A, C, E, G) or pBAV plasmids (B, D, F, H) were performed in minimal medium supplemented with either 100%, 50%, or 0% of the respective antibiotic. (A, B) Duration of lag phase, (C, D) maximum growth rate achieved, and maximum optical density reached (E, F) during 24 h of growth. (G, H) The proportion of antibiotic-resistant cells (i.e. a measure for plasmid loss) was determined by dividing the number of colonies formed on antibiotic-containing plates with the number of colonies formed on antibiotic-free agar plates after 24 h of growth under the respective conditions. Asterisks indicate significant differences between cells containing AT-rich and GC-rich plasmids within the same treatment. Independent-samples t-test: *** P < 0.001, ** P < 0.01, * P < 0.05, # P = 0.071, § P = 0.054, n = 8. (TIF) [file pgen.1007778.s002.tif]

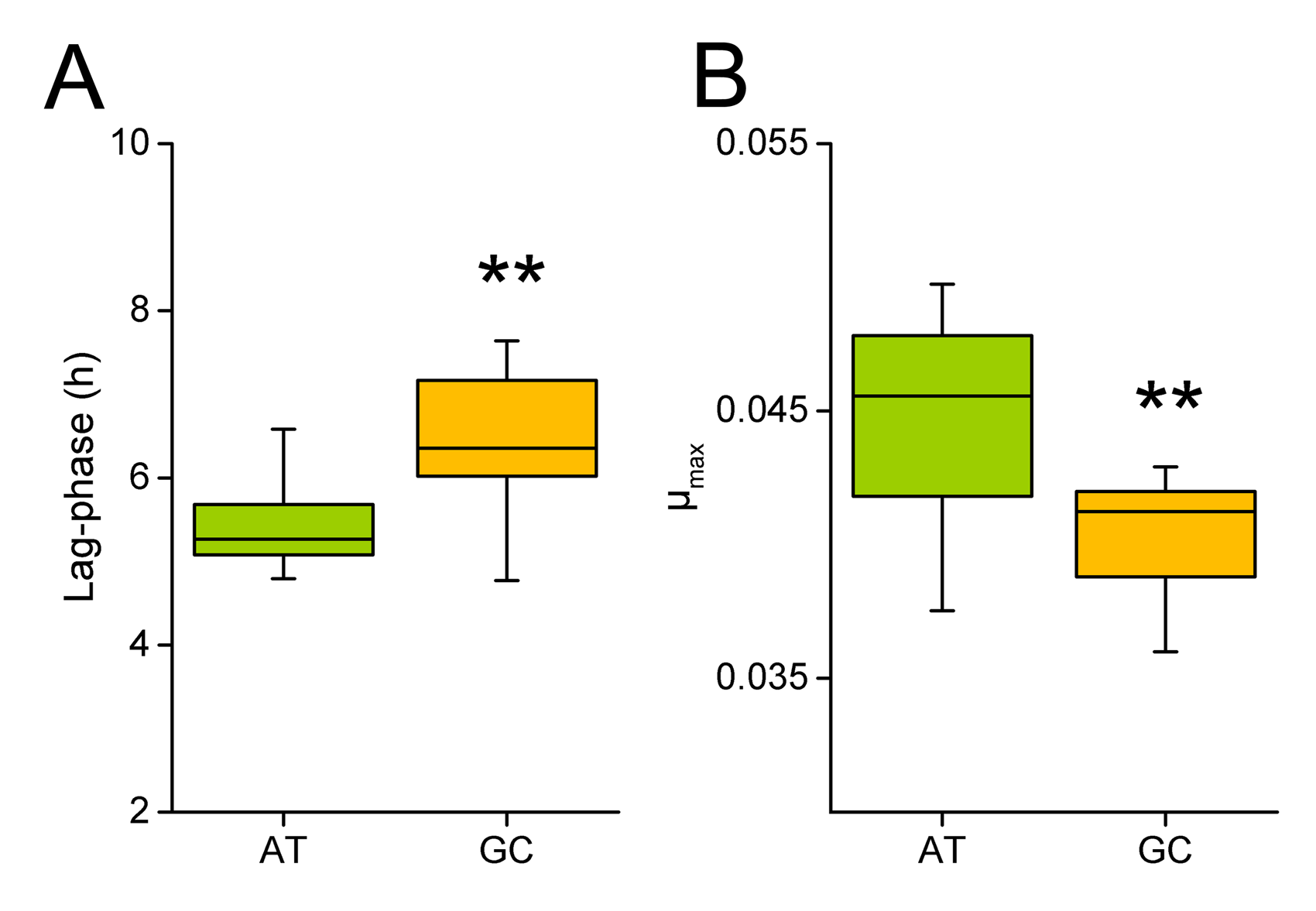

Supplement: S3 Fig — Growth experiments of E. coli Δhns harbouring AT-rich (green) or GC-rich (orange) pJet plasmids were performed in minimal medium. Growth over 24 h measured as optical density at 600 nm was used to calculate fitness-relevant parameters. (A) Duration of lag phase, (B) maximum growth rate. Asterisks indicate significant differences between cells containing AT-rich and GC-rich plasmids. Independent-samples t-test: ** P < 0.01, n = 16. (TIF) [file pgen.1007778.s003.tif]

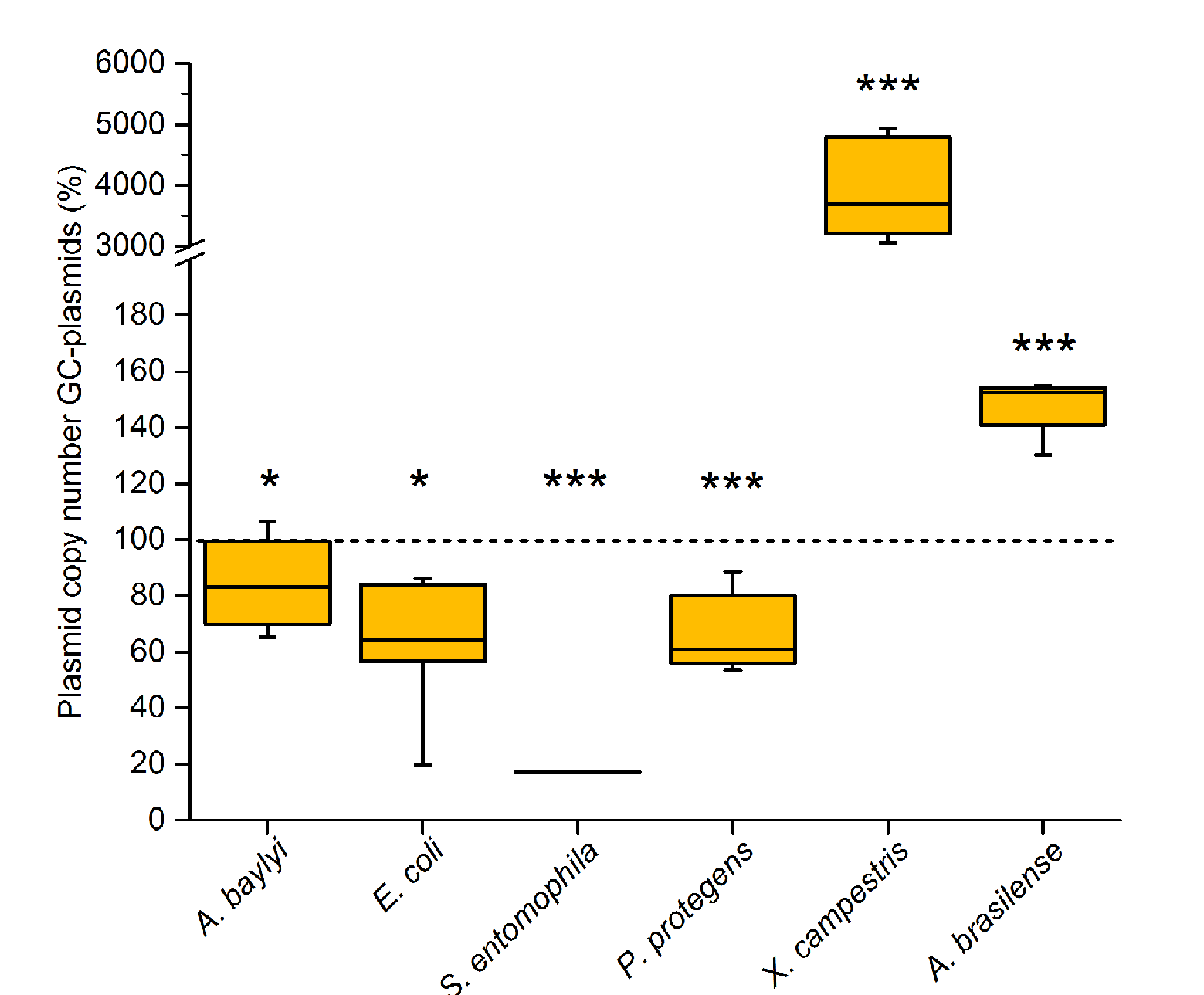

Supplement: S4 Fig — Copy number of GC-rich plasmids relative to AT-rich plasmids in the same bacterial species are displayed. Plasmid copy number per cell equivalent was assessed by quantitative real-time PCR and flow cytometry of all bacterial species after 24 h of growth. Asterisks denote significant deviations from equal copy numbers of AT-rich and GC-rich plasmids in the same host environment. One sample t-test: *** P < 0.001, ** P < 0.01, * P < 0.05, n = 5. (TIF) [file pgen.1007778.s004.tif]
