# Supplementary material for: Selective advantages favour high genomic AT-contents in intracellular elements
Source: PLoS Genet. 2019 Apr 29;15(4):e1007778. doi: 10.1371/journal.pgen.1007778 (PMC6519830; doi:10.1371/journal.pgen.1007778)
Supplement: S1 Table — AT-rich sequences (AT01-08) were amplified from the genome of Arabidopsis thaliana (chromosome 4), whereas GC-rich sequences (GC01-GC08) were amplified from Chlamydomonas reinhardtii (+chromosome 1, *chromosome 2). (DOCX) [file pgen.1007778.s005.docx]

**Supporting Information**

**Tables**

**Table S1. Non-coding AT- and GC-rich sequences used in this study.** AT-rich sequences (AT01-08) were amplified from the genome of *Arabidopsis thaliana* (chromosome 4)*,* whereas GC-rich sequences (GC01-GC08) were amplified from *Chlamydomonas reinhardtii* (^+^chromosome 1, ^*^chromosome 2).

| Sequence name | Length (bp) | GC-content (%) | Sequence position within genome | Primer sequences  (5’-3’) |
| --- | --- | --- | --- | --- |
| AT01 | 1,054 | 17.7 | 4,145,887 | for: AATCTATGTTTGTCAAGTGTTAG  rev: TGTGAGTTGTATATGTTTTGTTG |
| AT02 | 975 | 18.1 | 5,227,639 | for: TCTAGATTTTAACTCATTGAACTG  rev: TGAAGTTTGGTCCATATTAAAAC |
| AT03 | 962 | 19.3 | 1,095,268 | for: TATACAAATTAAAGAAAGCCAGC  rev: AATTCTCAAAACTGTACTAAGAG |
| AT04 | 1,010 | 21.9 | 1,956,554 | for: CAAAACCGAATGAATAACATAAC  rev: ACAATCTCTTGATTTTATCATGG |
| AT05 | 965 | 23.1 | 2,527,446 | for: ATTCTTTCTCTTGCTTATTCAAC  rev: ATTGATTACAATTCAACCTGTAG |
| AT06 | 960 | 24.5 | 313,339 | for: AATATACCAAGAGGTTTTGAAAG  rev: ATTTATACAAATGTGAGTGAGTG |
| AT07 | 978 | 19.9 | 6,808,034 | for: TACTTTCCGTTTAATCTGATTTC  rev: TTTCTTGTAATCTAGTGTTTAGC |
| AT08 | 1,021 | 20.7 | 8,729,507 | for: TAGATCCAATAAGCCCTATAATC  rev: TGCTTGAGTTAAATTTCATTTTG |
| GC01 | 1,032 | 85.3 | 5,762,266^+^ | for: AGGAGACGGCGGAGCAAGCAATG  rev: CCCAGCCCCGCAACTTCCAG |
| GC02 | 926 | 78.4 | 131,592^+^ | for: GGAGGTGGAGGTGGAGGCAGAG  rev: CGGCGTCAGCAGGAGCGATTTC |
| GC03 | 935 | 78.0 | 4,848,100^+^ | for: CTGCGCAGCAGGCCCAAACAG  rev: GACGTCTCAGCCCCCGTCTGTG |
| GC04 | 1,040 | 76.3 | 4,830,042^+^ | for: TCTGAACTCCGTAGGGCGTGACC  rev: GGCGGAGATGGCTGCGATTATGG |
| GC05 | 1,053 | 79.9 | 2,471,777^+^ | for: CTGCTGGCTTGCCTCAAGGACAC  rev: CACGTGCACGAAGCCGTACATGG |
| GC06 | 954 | 77.6 | 851,088^+^ | for: GCACCGTCGGAGTCGGGC  rev: CTTGCGGATCAGCGCCTCCAC |
| GC07 | 957 | 76.2 | 1,033,328^+^ | for: ACCACAGCGATGGCGGCTTG  rev: GTCCGCTCAGCGCATCTGATACC |
| GC08 | 1,029 | 75.9 | 416,710* | for: CTCACCTGTCAGCTCCACGATGC  rev: GAGCTGAAGGCGGCTGTGAAGG |

Abbreviations: for = forward primer, rev = reverse primer.
