# Supplementary material for: Selective advantages favour high genomic AT-contents in intracellular elements
Source: PLoS Genet. 2019 Apr 29;15(4):e1007778. doi: 10.1371/journal.pgen.1007778 (PMC6519830; doi:10.1371/journal.pgen.1007778)
Supplement: S2 Table — (DOCX) [file pgen.1007778.s006.docx]

**Supporting Information**

**Tables**

**Table S2. Primer pairs used for quantitative real-time PCR to determine the copy number of pJet and pBAV plasmids relative to the copy number of the chromosome.**

| Primer pair | target DNA element | target gene | 5’-3’ sequence | Reference | |
| --- | --- | --- | --- | --- | --- |
| dxs for/rev | Chromosome  *E. coli* | *dxs* | for: CGAGAAACTGGCGATCCTTA  rev: CTTCATCAAGCGGTTTCACA | [39] | |
| amp for/rev | Plasmid (pJet) | *bla* | for: CTACGATACGGGAGGGCTTA  rev: ATAAATCTGGAGCCGGTGAG | | [39] |
| kan for/rev | Plasmid (pBAV) | *aph(3’)* | for: GCATCAGGCTCTTTGACTCC  rev: CATCGGCCAGATCGTTATTC | This study | |
